# Nutri-Score and Nutrition Facts Panel through the Eyes of the Consumer: Correct Healthfulness Estimations Depend on Transparent Labels, Fixation Duration, and Product Equivocality

**DOI:** 10.3390/nu13092915

**Published:** 2021-08-24

**Authors:** Saar Bossuyt, Kathleen Custers, José Tummers, Laura Verbeyst, Bert Oben

**Affiliations:** 1 Smart Organizations, UCLL University of Applied Sciences, Geldenaaksebaan 335, 3001 Leuven, Belgium; saar.bossuyt@ucll.be (S.B.); jose.tummers@ucll.be (J.T.); 2 Health Innovation, UCLL University of Applied Sciences, Herestraat 49, 3000 Leuven, Belgium; laura.verbeyst@ucll.be; 3 Faculty of Arts, University of Leuven, Blijde Inkomststraat 21, 3000 Leuven, Belgium; bert.oben@kuleuven.be

**Keywords:** nutritional labeling, healthfulness perception, eye-tracking

## Abstract

Research on front-of-pack labels (FOPLs) demonstrated that Nutri-Score is one of the most promising FOPLs regarding healthfulness estimation accuracy. Nevertheless, as consumers are exposed to both the Nutri-Score and the mandatory Nutrition Facts Panel (NFP) in the supermarket, it is key to understand if and how both labels interact. This study investigates the contribution of Nutri-Score and NFP regarding healthfulness estimation accuracy, whether this impact differs depending on the product, and what role visual attention plays. We set up an eye-tracking experiment in a controlled setting in which 398 participants rated the healthfulness of 20 products. The results confirmed the positive impact of the Nutri-Score on healthfulness estimation accuracy, though the impact was larger for equivocal (i.e., difficult to judge) products. Interestingly, NFP either had no effect (compared to a package without Nutri-Score or NFP) or a negative effect (compared to a package with Nutri-Score alone) on healthfulness estimation accuracy. Eye-tracking data corroborated that ‘cognitive overload’ issues could explain why consumers exposed to Nutri-Score alone outperformed those exposed to both Nutri-Score and NFP. This study offers food for thought for policymakers and the industry seeking to maximize the potential of the Nutri-Score.

## 1. Introduction

The global prevalence of obesity has increased to pandemic rates over the last five decades [1,2,3]. Worldwide more than 1.9 billion of 18+ adults suffered from overweight in 2016, and more than one third of these were obese [4]. Given that poor diet quality is one of the most important modifiable risk factors for obesity and non-communicable diseases [5,6], the World Health Organization (WHO) repeatedly emphasizes the importance of taking measures that encourage populations to make healthier food choices. To accomplish this, consumers have to identify which products are the healthier ones and which are the less healthy ones. Hence, it is crucial that consumers are well informed about the nutritional quality of food products [7]. A key intervention to inform consumers and improve the healthfulness of consumers’ diets is nutritional labeling [8]. For many years the Nutrition Facts Panels (NFP), usually appearing on the back or side of the package, provide mandatory nutritional information about products [9]. However, extensive research has shown that the NFP contains technical and numerical information, which is perceived as complex and confusing and makes the information too effortful for consumers to process [10,11,12].

To complement the mandatory NFP, a growing number of countries are introducing front-of-pack (FOP) nutrition labels in which the information is easily accessible to consumers. FOP labels (FOPLs) provide simple, clear, and readily available information [13,14,15], which allows consumers to make rapid comparisons of the nutritional quality of products [10]. These labels are probably more suitable for shopping situations where usually quick decisions are made [7]. Existing research indicates that consumers prefer a simplified format of nutrition information on the front of the package [11,12]. Moreover, previous studies have shown that consumers’ objective understanding of FOPLs increases as the cognitive processing of nutrition labels decreases [12].

An increasing number of FOP nutrition schemes are implemented internationally. As a result of this multiplicity of FOPLs, many differences exist regarding graphic design and algorithms used [16]. In Europe, more and more countries are implementing the Nutri-Score label, after it was first implemented in France. The Nutri-Score is based on the British Food Standards Agency’s nutrient profiling system (FSA-NPS), which allocates positive points for energy, total sugar, saturated fat, and sodium and negative points for fruits, vegetables, nuts, fibers, and proteins [17]. It is a summary indicator that provides an overall evaluation of the nutritional quality of packaged food products, grading them on a scale of five letters/colors, ranging from A (dark green, which stands for most healthy) to E (red, which stands for least healthy). A large body of research has shown that the Nutri-Score label appeared to be the most effective FOP label, outperforming other FOPLs, such as Multiple Traffic Lights (MTL), Health Star Rating (HSR), and Guideline Daily Amounts (GDA), in helping consumers to correctly identify the healthfulness of food products [11,18,19,20,21,22,23,24,25,26,27,28]. In terms of the impact of FOPLs on purchase intentions, a study using a randomized controlled trial in a virtual web-based supermarket showed that of all FOPLs (GDA, MTL, Nutri-Score, Green Tick), the Nutri-Score label was the most effective in guiding consumers toward healthier food choices. Moreover, exposure to the Nutri-Score led to decreased sodium, lipids, and saturated fatty acids in the shopping basket [19]. In addition, Crosetto et al. [20] found that of five FOPL (MTL, RI’s, HSR, Nutri-Score, and Système d’Etiquetage Nutritionnel), the Nutri-Score had the greatest impact on improving the nutritional score of shopping baskets, followed by the HSR and MTL. Similarly, Dubois et al. [29] found, based on a real-life grocery shopping experiment, that the Nutri-Score was most effective and led to an increase in the purchase of high nutritional quality and a decrease in medium and low nutritional quality products. The Nutri-Score enabled participants to improve the nutrition quality of the purchased food by 2.5% [29]. Poquet et al. [30] further demonstrated that this not only holds for adults but also for children: Both mothers and their children appear to make healthier food choices (e.g., for afternoon snacks) when the products are provided with Nutri-Score labels.

Existing research on the impact of Nutri-Score on healthfulness estimation of products tends to focus on its relative impact compared to international FOPLs. There is, however, a lack of research on how Nutri-Score interacts with the mandatory NFP. In particular, it is unclear what the added value of each label (individually and combined) is in terms of helping consumers make better healthfulness estimations. We want to further unravel whether Nutri-Score labels might suffice for consumers to make correct estimations, or whether they (for some products or product categories) rather look for more information in the NFP, or whether the combination of Nutri-Score and NFP (which is an increasing reality consumers are faced with) boosts correct estimations even further or rather confuses consumers and leads to an information overload (considering that NFP labels have been demonstrated to be complex and difficult to process).

In most previous research on the objective understanding of the Nutri-Score, participants were exposed to mock product packs, showing only the front of the pack and not the back/side with NFP [16,19,23,25,26,31]. However, in reality, consumers may look at both the front (with the Nutri-Score) and the back/side (with the NFP) of a pack when assessing the healthfulness of products. Therefore, in the current study, we create four experimental conditions in which participants can be exposed to product packaging with (1) no Nutri-Score and no NFP, (2) Nutri-Score but no NFP, (3) NFP but no Nutri-Score, or (4) both Nutri-Score and NFP. This design enables us to examine the exact contribution of each labeling system to consumers’ ability to estimate the healthfulness of products. Thus, the first research question (RQ) is formulated as follows:

RQ1: “What is the impact of the Nutri-Score label and the NFP on the healthfulness estimation of products?”

In addition, the current study will investigate whether the impact of the Nutri-Score and the NFP on healthfulness estimation is the same for products from different categories and with different Nutri-Score grades (A-B-C-D-E). Concerning product categories, in existing research, the focus is nearly exclusively on products that are inherently categorized as unhealthy, such as cake, breakfast cereals, and pizza [16,25,26,27,31,32,33,34,35]. To implement adequate policy strategies that improve consumers’ diets effectively, it is important to extend the insights into the impact of Nutri-Score on the healthfulness perceptions to a wider range of products. To address this, we will include 20 products from four frequently bought, yet under-researched product categories (beverages, ice cream, prepared meals, and dairy products) in our study.

Next, it is important to assess whether the Nutri-Score and the NFP affect the healthfulness perceptions of products with different Nutri-Score grades in the same way. With regards to the impact of the Nutri-Score, there are some signs that certain color codes might affect healthfulness perceptions more than others. For instance, in an online choice experiment on the impact of MTL, researchers found that food products that contained more green lights were 6.1 times more likely to be labeled as healthy, whereas products with more red lights were 11.4 times less likely to be labeled as healthy [36]. In general, studies on the impact of MTL labels found that red lights tend to outweigh the green lights when consumers are estimating the healthfulness of virtue (i.e., healthy) products [28]. In a recent study, De Temmerman et al. [22] also looked into the impact of traffic lights colors indirectly by considering the impact of the different Nutri-Score grades (A-B-C-D-E) on the perceived healthfulness of food products. The presence of the Nutri-Score interacted significantly with the Nutri-Score grade. In contrast with previous research that demonstrated a higher impact of red-colored FOP labels [28,36], De Temmerman et al. [22] found that the impact of the Nutri-Score presence on perceived healthfulness was only present in products with a Nutri-Score grade A (dark green) or B (light green). For products with Nutri-Score C (yellow), D (orange), and E (red), no significant interaction effects with the presence of the Nutri-Score were found [22]. In sum, there seems to be conflicting evidence concerning the impact of green, orange, and red colors in FOP labels on healthfulness perceptions of products. This highlights the importance again to include a balanced set of products from different Nutri-Score grades in the stimuli set, which brings us to the second research question of the present study:

RQ2: “Is the impact of the Nutri-Score label and the NFP on healthfulness estimation different for different types of products?”

Finally, even though visual attention to nutrition labels is key to process and comprehend the information, only a limited number of studies assessed this criterion [37]. Previous research mainly focused on two topics. First, a couple of studies explored how consumers divide their attention across FOPL and NFP. The results showed that when consumers are exposed to both traffic light colored FOPLs and NFP, the former received more visual attention than the latter [8,35,36]. More specifically, FOPLs are viewed more rapidly [10,37] and more often than the NFP [37]. Furthermore, attention to the more comprehensive information on the NFP has been shown to diminish when FOPLs are present as well. Instead of steering consumers’ attention to the information on the NFP, FOPLs serve more as a cognitive shortcut to nutrition information [10,28].

Second, other eye-tracking studies focused on the impact of colors on visual attention. It was found that the presence of color coded FOPLs increased attention to the FOP label [37], and, in fact, attention to any type of nutrition information (either FOP or NFP) [10]. This is supported by Koenigstorfer et al. [38], who found that visual attention to product packages increased when traffic light color-coded FOPLs were displayed on the packages. Additionally, Jones and Richardson [39] compared the visual attention to specific nutrients on general NFPs with NFPs accompanied by a traffic light color-coded food label. They found that the traffic light color-coded label facilitated participants in guiding their attention to the important nutrients. The nutrients that gained more visual attention were also used more frequently in rating the healthfulness of the product [39].

So far, there is no research that has examined the impact of visual attention to FOPLs in general and the Nutri-Score label, in particular, on consumers’ ability to correctly estimate the healthfulness of food products, let alone the interaction with visual attention to NFP (cf. RQ1). The current study will, therefore, add to existing research by relating visual attention to the Nutri-Score label and to the NFP on consumers’ performance in estimating the healthfulness of food products. The third research question is, therefore, formulated as follows:

RQ3: “Does more visual attention for Nutri-Score and/or NFP make consumers’ healthfulness estimations more accurate?”

## 2. Materials and Methods

### 2.1. Design and Stimuli

To provide an answer to the research questions, we developed an exercise in which participants had to estimate how (un)healthy 20 products were on a scale from 1 (unhealthy) to 5 (healthy). For each product, we calculated the absolute difference between the healthfulness estimation of the participant and the true health score of the product (i.e., the Nutri-Score, where we considered Nutri-Score grade A as 5 and Nutri-Score grade E as 1 on the scale from unhealthy (1) to healthy (5)). Based on this information, the ‘average mistake’ (AM) participants made across 20 products was calculated:

(1) $$\mathrm{AM}=\frac{\sum_{i=1}^{20}\left|Participant^{\prime }s\;estimation\;for\;product\;i-NutriScore\;grade\;of\;product\;i\right|}{20}$$

In sum, a high score on AM implied bad performance on the healthfulness estimation exercise, whereas a low score on AM implied good performance.

All participants were exposed to the same 20 product visuals (see Table A1 in Appendix A), all of which showed real, private label products from a large retailer. The main reason to work with real instead of mock products, as is common in many studies on the impact of FOP labels, was ecological validity: We aimed to enable more accurate predictions of how Nutri-Score and NFP affect healthfulness estimations in reality (i.e., with actual products). Furthermore, the products were selected to create a balanced design of categories and Nutri-Score grades: There were four product categories (beverages, ice cream, prepared meals, and dairy products), and within each category, the five Nutri-Score grades (A-B-C-D-E) were represented once. The order in which product categories were shown was randomized, and within the categories, the order of the products (and thus, the different Nutri-Score grades) was also randomized. The main reason not to opt for a fully randomized design, mixing categories and products, was again ecological validity: We aimed to mimic the situation in the supermarket where consumers are exposed to shelves organized per category, which compares the healthfulness of products within categories.

While all participants assessed the same set of products, the product visuals they were exposed to were slightly different depending on the experimental condition participants were assigned to. More specifically, there were two experimental manipulations (Nutri-Score presence and NFP presence) that determined whether the product visual featured a Nutri-Score label and/or was accompanied by an NFP. The experimental design is exemplified in Table 1.

### 2.2. Procedure

Participants were welcomed and briefed by a member of the experiment team. In the meantime, another member of the team allocated an anonymous respondent number to the participant, which would be used later to connect eye-tracking data to survey data. This team member also assigned the participant at random to one of the four experimental conditions (see Table 1). Upon completion of the informed consent, participants were invited to sit down in front of a computer screen with a Tobii Pro X2 eye tracker mounted to it. This binocular device records gaze data at a 60 Hz frequency (i.e., one data point for each eye every 16.66 ms). The built-in fixation filter, Tobii I-VT [40], transformed those raw data into fixation events. First, a member of the team guided them through the calibration procedure in Tobii Studio (eye-tracking software), and entered their respondent number. Next, participants completed the healthfulness estimation exercise, in which they had to rate the healthfulness of 20 products on a scale from 1 (unhealthy) to 5 (healthy), while their eye movements were being tracked. Upon completion of this exercise, participants received an instruction to notify a member of the experiment team. Subsequently, they were guided to a different computer (without an eye-tracking device mounted to the screen) to complete the last part of the experiment, viz. a survey. In this final survey, participants answered questions on variables that could influence their capability to make correct healthfulness estimations, such as sociodemographic variables and lifestyle variables (such as nutrition knowledge, self-estimated diet quality, nutrition behavior, and grocery shopping behavior). We also included a variable “Nutri-Score familiarity”, which measured the degree to which participants are familiar with the Nutri-Score concept, since the Nutri-Score label has only been introduced (on a voluntary basis) in Belgium in April 2019. When they completed the final survey, participants notified a member of the team who guided them back to the exit. If participants wanted to, they could enter their e-mail address in a separate database to take part in a lottery to win a €20 gift card in exchange for their participation.

### 2.3. Participants

Over one week, 409 people took part in our study. Participants were recruited in two different locations: Our primary experiment location was a university campus in the city center of Leuven, a mid-sized city in the Dutch-speaking part of Belgium. In this location, participants were recruited both on and off campus (in the city center), which allowed for a more diverse sample regarding educational and professional background. Our secondary location was the campus of a university of applied sciences, outside the city center. Since the study dealt with healthfulness perceptions of products in the supermarket and how Nutri-Score and NFP can affect this, we determined beforehand to exclude people who never shop for groceries. Eleven people did not meet this requirement, and were, hence, eliminated from the sample. In addition, due to technical issues with the eye-tracking equipment and/or issues with calibration, twelve participants completed the healthfulness estimation exercise without eye-tracking. This left us with two samples: A general sample of 398 participants, who successfully completed the healthfulness estimation exercise (with or without eye-tracking) and the subsequent questionnaire, and an eye-tracking sample of 386 participants who successfully completed the healthfulness estimation exercise with eye-tracking and the subsequent questionnaire. The main sociodemographic characteristics of the general sample can be found in Table 2. Overall, there were no major differences between the cells regarding sociodemographic characteristics. The significant differences for ‘Expertise in health’ and ‘Following a weight loss diet’ were most likely caused by the small number of participants answering ‘yes’ to those criteria.

In addition, we also investigated whether food knowledge and reported diet behavior were equal in all the cells. To this end, we ran a one-way ANOVA to compare the mean scores on three constructs (see Table A2 in Appendix B for more details on the items): Nutrition Knowledge (α = 0.71), Self-Estimated Diet Quality (α = 0.89), and Nutrition Behavior (α = 0.60). As shown in Table 3, there were no significant differences, indicating that participants in all experimental conditions had highly similar levels of food knowledge and reported diet behavior. This applies to BMI too: There were no statistically significant differences between group means.

### 2.4. Statistical Analyses

As illustrated higher in Section 2.1, our study had a crossed effects design, with all subjects participating in the experiment responding to the same set of stimuli (i.e., 20 products). As a consequence, the resulting data display a twofold violation of the assumption of independence of observations underlying traditional linear models, since the data are both grouped under the participants (each participant scoring 20 stimuli in one of the four experimental conditions) and the stimuli (each stimulus being scored by 398 participants in one of the four experimental conditions). In other terms, the scores by one participant, as well as the scores for one stimulus, will tend to show a degree of homogeneity and differ from the scores by other participants and those for other stimuli. To deal with this nested data structure, mixed-effects models (also known as a multilevel model or hierarchical model) were fitted [41,42,43,44,45].

Mixed-effects models combine fixed and random effect terms: Fixed effect terms exhaust all levels of a parameter, their values covering all values in the population; random effect terms are sampled from a larger population, and therefore, only represent a sample of the actual population and are modeled as random variables with 0 as mean and an unknown variance (N(0,σ²)). In mixed-effects models, the non-independence of data is accounted for by the random effect terms representing the grouping variables, viz. the participants and stimuli (20 products) in the present experiment. These random effects can take the form of random intercepts, as well as random slopes. Assuming a common slope for the fixed effects (or covariates), random intercepts estimate separate intercepts for each value of a grouping variable, modeling the deviation of their variance to the overall variance represented by the fixed intercept. In models with random intercepts and random slopes, the slope of a fixed effect term is allowed to vary based on the grouping variable. From an analytical perspective, random slopes for a fixed effect term amount to an interaction between the random effect term (grouping variable) and the fixed effect term (covariate).

### 2.5. Eye-Tracking Analyses

As explained earlier in Section 2.2, we used Tobii Studio for eye-tracking recording and analysis. Since we were mainly interested in visual attention for the Nutri-Score label on the product and/or the NFP under the product, we focused on two key metrics: Fixation count (or the number of fixations on Nutri-Score and/or NFP) and total fixation duration (or the length of the fixations on Nutri-Score and/or NFP). To account for differences in pack size (and thus, Nutri-Score label size), and NFP size, we worked with relative, instead of absolute, measures:

(2) $$\mathrm{Relative}\;\mathrm{fixation}\;\mathrm{count}\;\mathrm{Nutri}-\mathrm{Score}=\frac{Number\;of\;fixations\;on\;Nutri-Score\;label}{Number\;of\;fixations\;on\;package\;as\;a\;whole}$$

(3) $$\mathrm{Relative}\;\mathrm{fixation}\;\mathrm{duration}\;\mathrm{Nutri}-\mathrm{Score}=\frac{Length\;of\;fixations\;on\;Nutri-Score\;label}{Length\;of\;fixations\;on\;package\;as\;a\;whole}$$

(4) $$\mathrm{Relative}\;\mathrm{fixation}\;\mathrm{count}\;\mathrm{NFP}=\frac{Number\;of\;fixations\;on\;NFP}{Number\;of\;fixations\;on\;package\;as\;a\;whole}$$

(5) $$\mathrm{Relative}\;\mathrm{fixation}\;\mathrm{duration}\;\mathrm{NFP}=\frac{Length\;of\;fixations\;on\;NFP}{Length\;of\;fixations\;on\;package\;as\;a\;whole}$$

As these measures were highly correlated, both for Nutri-Score (*r*(194) *=* 0.98, *p* < 0.001) as for NFP (*r*(185) *=* 0.99, *p* < 0.001), we decided to only run analyses on one of them. We picked Relative Fixation Duration, as previous eye-tracking research found this to be the most reliable measure of attention [46,47].

## 3. Results

### 3.1. The Impact of the Nutri-Score Label and the NFP on the Healthfulness Estimation of Actual Products

The full model (see Table A3 in Appendix C), including all sociodemographic variables (see Table 2), as well as the variables measuring food knowledge and reported diet behavior (see Table 3), revealed that the inclusion of most terms did not result in a significant reduction of the unexplained variance. The best-fitting model explaining the average mistake (AM) in estimating the healthfulness of products was obtained by the following linear mixed-effects model (*r*² = 0.42):

- Random intercept for participants and stimuli (products);
- By-stimulus random slopes for both experimental manipulations (Nutri-Score and NFP);
- Fixed effects for the experimental manipulations (Nutri-Score and NFP) and their interaction (Nutri-Score × NFP), for age and Nutri-Score familiarity (see Table 2 for scale items).

We first inspected the fixed part of the model as summarized by the output of a Type II Wald *χ*² test in Table 4 [48].

**Figure 1:**
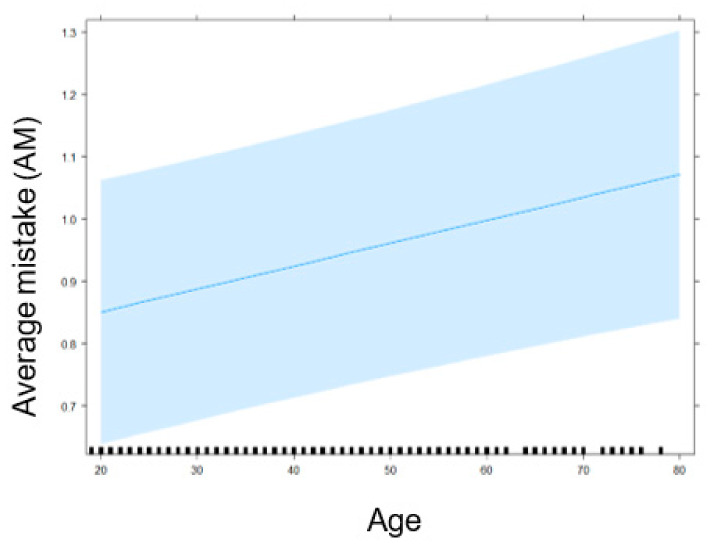
Impact of Age on AM in the healthfulness estimation exercise.

**Figure 2:**
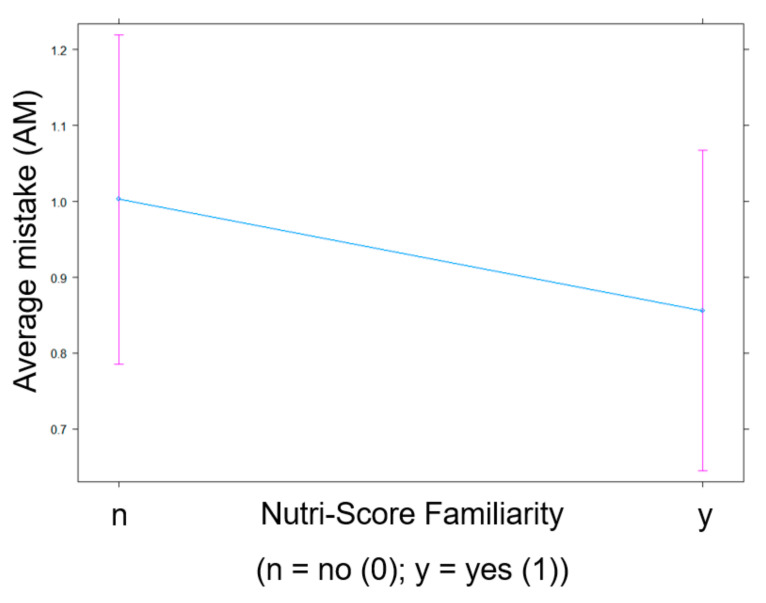
Impact of Nutri-Score Familiarity (n = no, not (very) familiar (0); y = yes, (very) familiar (1)) on AM in the healthfulness estimation exercise.

**Figure 3:**
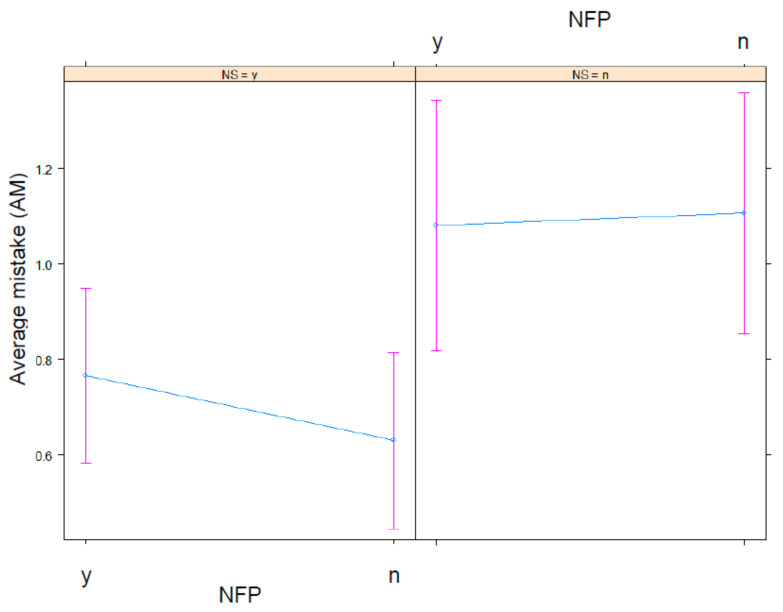
Impact of Nutri-Score (NS: n = no (0); y = yes (1)) and NFP presence (n = no (0); y = yes (1)) on AM in the healthfulness estimation exercise.

The significant fixed effects are visualized in several plots. Figure 1 identifies a participant’s higher age as indicative of their erroneous scoring of a product’s healthfulness. Stated more simply: Older participants make more mistakes. Next, Figure 2 illustrates that participants who know and understand Nutri-Score make fewer mistakes in estimating the healthfulness of products than participants who did not display that knowledge. As for the interaction between Nutri-Score and NFP, Figure 3 displays a higher AM score in the experimental conditions without Nutri-Score than the conditions with Nutri-Score. This indicates that the presence of Nutri-Score on products helps participants to make more accurate healthfulness estimations. Furthermore, it is interesting to note that, in the conditions without Nutri-Score, NFP presence did not affect AM at all. Adding NFP to a product pack (without Nutri-Score) is, thus, not helpful. In addition, we observed a slight increase of the AM in the condition where both Nutri-Score and NFP are present (compared to the condition where only Nutri-Score is present), suggesting a potential information overload in that condition.

To conclude, regarding our first research question (RQ1), our findings demonstrate that the Nutri-Score has a positive impact on healthfulness perception of products, while this effect is either non-existing or slightly negative for NFP.

### 3.2. The Impact of the Nutri-Score Label and the NFP for Different Types of Products

To answer RQ2, we turn to the random part of the model. First, we analyzed the intraclass correlation coefficient (ICC) of the random effect terms. This statistic can be interpreted in two complementary ways: First, it quantifies the share of the variance accounted for by the random effect term; second, it gauges the internal cohesion between the data within the clusters formed by the random effect term. Table 5 summarizes the ICC of the random effect terms, significantly reducing the unexplained variance.

**Figure 4:**
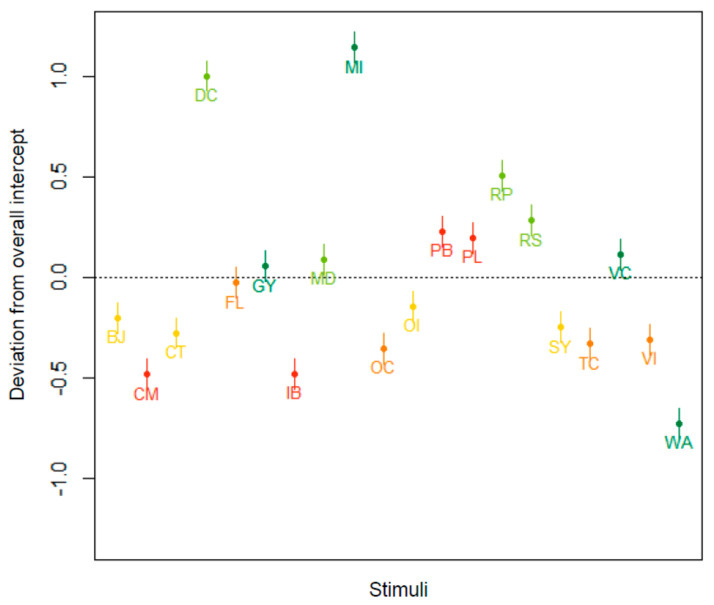
By-stimulus random intercepts for the 20 products used as stimuli in the experiment. WA = water; DC = diet coke; BJ = blood orange juice; FL = fresh lemonade; PL = processed lemonade; MI = mocha ice cream; RS = raspberry sorbet; OI = orange ice cream; VI = vanilla ice cream; IB = ice cream bars; VC = vegetable curry; MD = meat dish; CT = chicken tajine; TC = Thai curry; PB = prawns in garlic butter; GY = Greek yoghurt; RP = rice pudding; SY = strawberry yoghurt; OC = organic chocolate mousse; CM = chocolate mousse.

The data in Table 5 suggest a considerable impact of the stimuli on the AM, explaining a quarter of the variance in the data. Figure 4 represents the random intercepts for the 20 products used as stimuli in the experiment. The colors correspond to the Nutri-Score grades (A = dark green; B = light green; C = yellow; D = orange; E = red), and the horizontal black dotted line is the fixed intercept, averaging the random intercepts. We clearly see that, irrespective of the fixed part of the model, certain products, such as mocha ice cream (MI) and diet coke (DC), induced a much higher AM, whereas other products, such as water (WA), induced a lower AM. In sum, these results demonstrate that certain products were more equivocal, posing a greater challenge for participants estimating the healthfulness than others.

**Figure 5:**
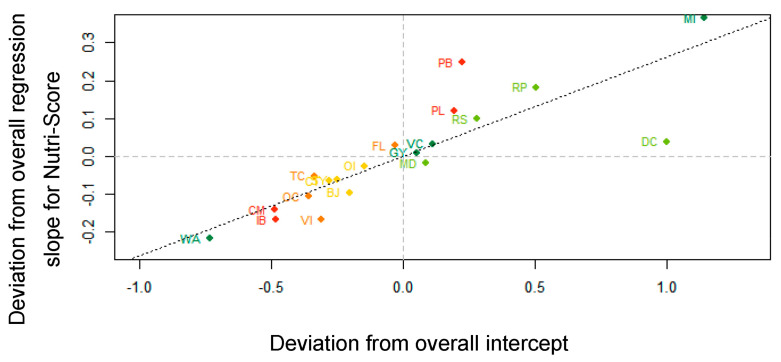
Correlation (*r* = 0.82) between by-stimulus deviations from overall intercept and by-stimulus deviations from overall regression slope for Nutri-Score. Abbreviations of the product names are explained in Figure 4 and in Appendix A (Table A1).

Next, three correlations between random effect terms justify a closer inspection. First, the strong correlation (*r* = 0.82) between the by-stimulus random intercepts and the by-stimulus random slopes for Nutri-Score suggests that the presence of the Nutri-Score label reduces the AM of nutritionally equivocal stimuli (see Figure 5). Notwithstanding a few exceptions, products with a negative random intercept (viz. with a lower AM in an empty model) have a random regression slope that is flatter than average (viz. with a lower impact on the reduction of the AM); conversely, products with a positive random intercept display a steeper slope for Nutri-Score, suggesting a higher impact of the presence of the Nutri-Score on the reduction of the AM. The stimuli where this effect is most outspoken, are prawns in garlic butter (PB), and to a lesser extent, mocha ice cream (MI), rice pudding (RP), and processed lemonade (PL). For diet coke (DC), the Nutri-Score label does not achieve its full potential. It is also noteworthy that, except for water (WA) in the bottom left quadrant (low AM, low Nutri-Score impact), all Nutri-Score grade A and B products are in or near the top right quadrant of the graph (high AM, high Nutri-Score impact). This suggests that while participants were clearly aware that water is healthy, they were much less confident about the healthfulness of other Nutri-Score grade A and B products. A similar effect seems to occur in Nutri-Score grade E products: Certain products, such as chocolate mousse (CM) and ice cream bars (IB), were probably intuitively perceived as unhealthy, while others, such as prawns in garlic butter (PB) and processed lemonade (PL), were more challenging.

**Figure 6:**
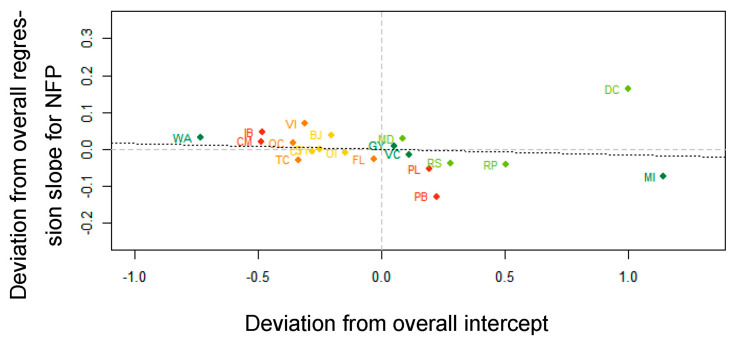
Correlation (*r* = −0.11) between by-stimulus deviations from overall intercept and by-stimulus deviations from overall regression slope for NFP. Abbreviations of the product names are explained in Figure 4 and in Appendix A (Table A1).

Second, although the correlation between the by-stimulus random intercepts and by-stimulus random slopes for NFP was rather weak (*r* = −0.11), it singled out diet coke (DC) as a product where NFP presence seemed to have a clear impact on AM (see Figure 6). The difference between diet coke (DC) and other products could be explained by the product’s easily interpretable NFP. More specifically, the NFP of diet coke consists of a text that states the product contains ‘zero of everything’ (0.2 calories, 0 fats, <0.1 carbohydrates, 0 fibers, 0 proteins, and 0.1 salt).

**Figure 7:**
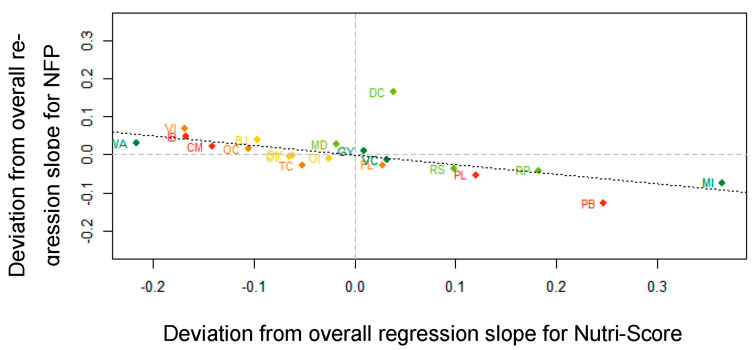
Correlation (*r* = −0.62) between by-stimulus deviations from overall regression slope for Nutri-Score and by-stimulus deviations from overall regression slope for NFP. Abbreviations of the product names are explained in Figure 4 and in Appendix A (Table A1).

Third, the negative correlation (*r* = −0.62; visualized in Figure 7) between the by-stimulus random slopes for NS and NFP identifies a complementary effect of both labeling systems used in the experiment, corroborating the findings for the Nutri-Score × NFP interaction in the fixed part of the model (see Table 4): For those products in which Nutri-Score was the most helpful in reducing AM, such as mocha ice cream (MI), prawns in garlic butter (PB), Rice Pudding (RP) and processed lemonade (PL), the NFP was the least helpful. Diet coke (DC) was again the most notorious exception to this tendency.

In sum, to answer our second research question (RQ2), the findings show that the impact of the Nutri-Score label and the NFP was indeed different for different types of products: The impact of the Nutri-Score was largest for products that are identified as equivocal (i.e., more difficult to judge), while the impact of the NFP was only significant for products with a clear and simple NFP.

### 3.3. The Impact of Visual Attention for Nutri-Score and/or NFP on Consumers’ Healthfulness Estimations

Since the availability of eye-tracking data was constrained by the specific experimental configuration to which a participant was subjected (see Table 1, the eye-tracking sample was split into two subsets: One for participants exposed to products with Nutri-Score (*n* = 196) and one for participants exposed to products with NFP (*n* = 187). These subsets overlap for participants (*n* = 96) exposed to products with both Nutri-Score and NFP. In the first subset, we explored whether AM was driven by visual attention for the Nutri-Score label, and whether this was different for participants exposed to Nutri-Score only versus participants exposed to both Nutri-Score and NFP. In the second subset, we investigated whether AM was driven by visual attention for NFP, and whether this was different for participants exposed to NFP only versus participants exposed to both Nutri-Score and NFP. For each subset, the following mixed-effects models were estimated to explain AM:

- Random intercept for the participants and the stimuli;
- By-stimulus random slopes for the experimental condition complementary to the one defining the subset (Nutri-Score or NFP present/absent) or for the relative fixation duration. For each random slope, a separate analysis was carried out because a model combining both random slopes resulted in a singular fit, due to overfitting;
- Fixed effect terms for the interaction of the experimental condition complementary to the one defining the subset (Nutri-Score or NFP present/absent) and the relative fixation duration

#### 3.3.1. Eye-Tracking Results in the Subset of Participants Exposed to Products with Nutri-Score

**Figure 8:**
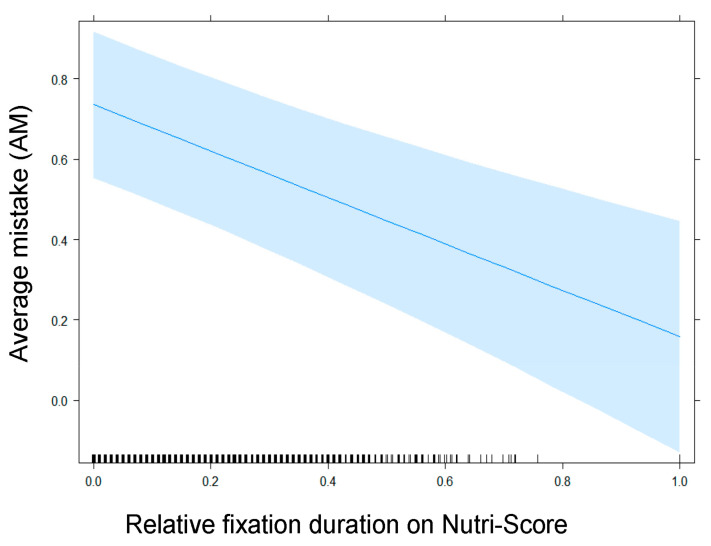
Impact of the relative fixation duration on Nutri-Score on AM in the subset of participants exposed to Nutri-Score.

Table 6 shows the fixed part of the first model with a random slope for NFP summarized using the Type II Wald *χ*² statistic. The only significant variable is Relative fixation duration on Nutri-Score: As shown in Figure 8, participants who paid more visual attention to the Nutri-Score label on the products performed better on the healthfulness estimation exercise (higher relative fixation time on Nutri-Score corresponds to a lower AM score).

**Figure 9:**
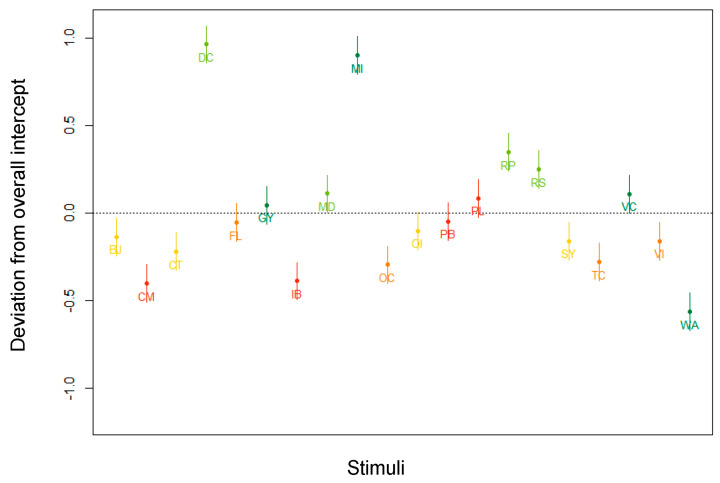
By-stimulus random intercepts in the subset of participants exposed to products with Nutri-Score. Abbreviations of the product names are explained in Figure 4 and in Appendix A (Table A1).

When we look at the ICC for the random effect terms in Table 7, the random intercepts clearly account for a major reduction of the variance. Figure 9 visualizes the by-stimulus random intercepts. It can be observed that, all other variables kept stable, participants had more trouble estimating the healthfulness of diet coke (DC) and mocha ice cream (MI) (higher AM), and less trouble estimating the healthfulness of water (WA) (lower AM). These results are in line with the ones generated by the full dataset.

The second model, with a random slope for the relative fixation duration on NS, could not be built because of overfitting.

#### 3.3.2. Eye-Tracking Results in the Subset of Participants Exposed to Products with NFP

The first model in this subset comprised Nutri-Score presence, relative fixation duration on NFP, and their interaction as fixed effect terms, by-participants and by-stimuli random intercepts, and a by-random slope for Nutri-Score presence. The fixed part of the model, summarized in Table 8, shows a significant impact of both main effects but not of their interaction.

**Figure 10:**
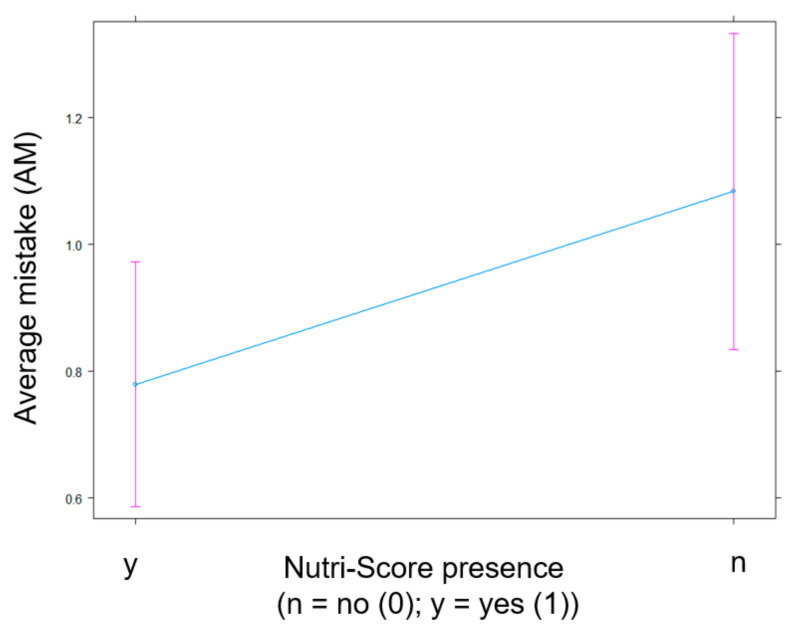
Impact of the presence of Nutri-Score (n = no (0); y = yes (1)) on AM in the subset of participants exposed to products with NFP.

The visualization of the main effect for Nutri-Score in Figure 10 corroborates the previously established reduction of the AM made by participants in the condition where the Nutri-Score label is present (see higher in Figure 3).

**Figure 11:**
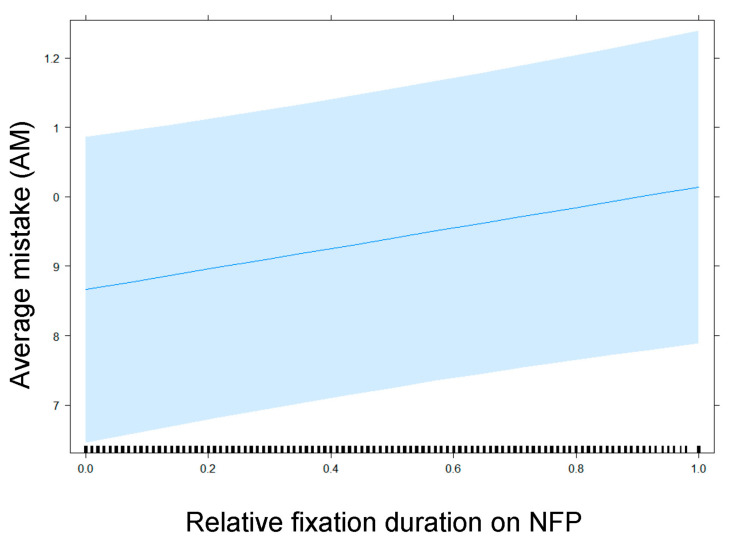
Impact of relative fixation duration on NFP on AM in the subset of participants exposed to products with NFP.

Furthermore, an increase of the relative fixation time on NFP correlates with the degree of AM made by the participants gauging the healthfulness of products (Figure 11): Interestingly, a higher relative amount of time spent looking at the NFP correlates with more mistakes.

The random part of the model, outlined in Table 9, unveils a substantial impact of the by-stimulus random intercepts.

**Figure 12:**
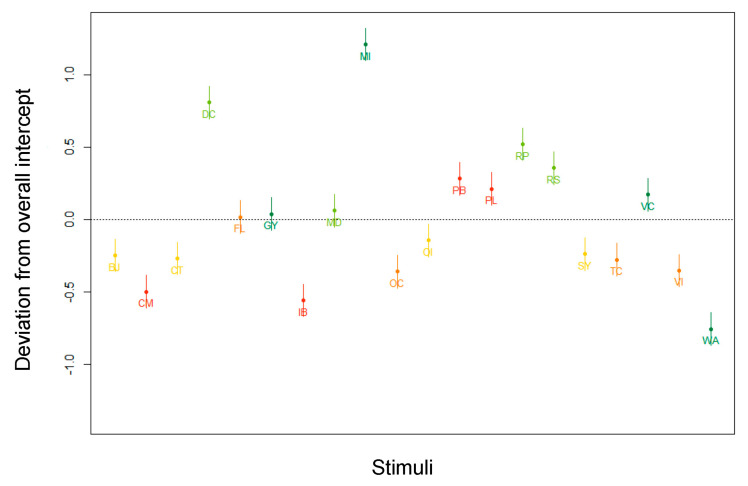
By-stimulus random intercepts in the subset of participants exposed to products with NFP. Abbreviations of the product names are explained in Figure 4 and in Appendix A (Table A1).

The by-stimulus deviations from the overall intercept in Figure 12 are in line with the findings of the parallel model built for the subset of participants exposed to products with Nutri-Score and the full subset: Diet coke (DC) and mocha ice cream (MI) clearly induce a higher degree of AM, whereas water (WA) produces an opposite effect.

**Figure 13:**
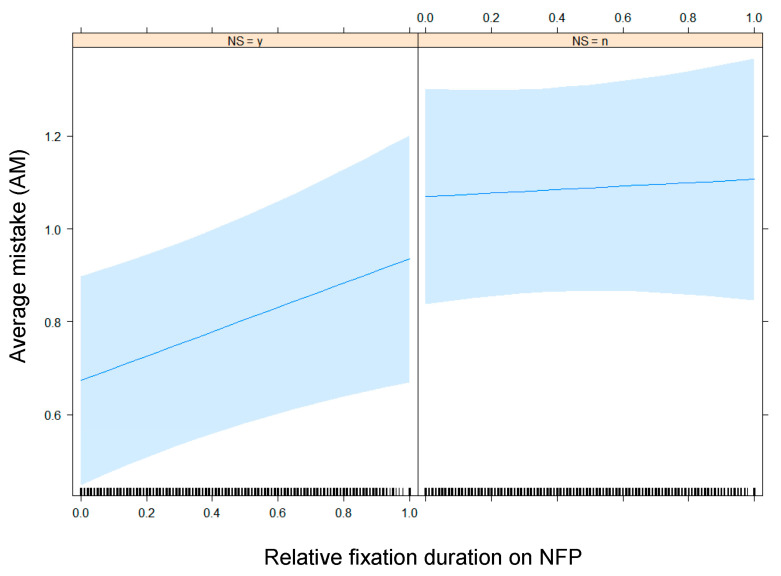
Impact of relative fixation duration on NFP on AM in the presence (NS = y or yes (1)) or absence (NS = n or no (0)) of the Nutri-Score label, in the subset of participants exposed to products with NFP.

The second model built for the subset consisting of the participants who were in the condition of products with NFP, is the model with Nutri-Score presence, relative fixation duration on NFP and their interaction as fixed effect terms, by-participants and by-stimuli random intercepts, and by-stimuli random slopes for the relative fixation duration on NFP. This model unveils a significant effect of Nutri-Score presence and a significant interaction between Nutri-Score presence and relative fixation duration on NFP (Table 10), visualized in Figure 13. Only in the condition where the participants were exposed to products with the Nutri-Score, as well as the NFP, did an increase of the relative fixation duration on NFP correspond with an increase of AM.

The random part of this model, outlined in Table 11, uncovers a major impact of the by-stimulus random slopes, in addition to the by-stimulus random intercepts.

**Figure 14:**
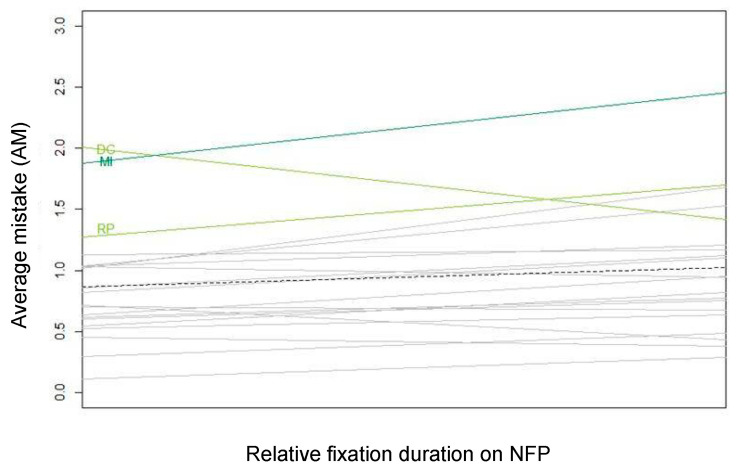
By-stimulus regression slopes for relative fixation duration on NFP in the subset of participants exposed to products with NFP. Abbreviations of the product names are explained in Figure 4 and in Appendix A (Table A1).

Figure 14 plots the by-stimulus random intercepts and slopes for relative fixation duration on NFP. The black dashed line is the fixed regression slope for relative fixation duration on NFP; the grey slopes are the by-stimulus regression slopes with some of them highlighted according to the Nutri-Score color scheme (A= dark green; B = light green; C = yellow; D = orange; E = red). Whereas longer relative fixation duration is on average indicative of a higher degree of AM (especially for products, such as mocha ice cream (MI) and Rice Pudding (RP), exemplified by a steeper than average slope), an opposite tendency can be observed for diet coke (DC), which constitutes an exception to the rule that more attention for NFP results in worse performance on the healthfulness estimation exercise. As previously established, diet coke (DC) has a more interpretable NFP, which explains why longer visual attention for the NFP resulted in a better performance on the healthfulness estimation exercise.

Regarding the third research question (RQ3), we demonstrated that visual attention for the Nutri-Score did indeed make consumers’ healthfulness estimations more accurate. Visual attention for the NFP, however, correlated with less accurate healthfulness estimations. The only exception to this latter finding was again diet coke, the only product with a simple NFP.

## 4. Discussion

This study investigated the (combined) impact of Nutri-Score and NFP labels on consumers’ ability to make accurate healthfulness estimations (RQ1), whether this impact is the same for different products (RQ2), and how visual attention for Nutri-Score and NFP affects all this (RQ3). In line with previous research, we found convincing evidence that the Nutri-Score label positively affects accuracy in healthfulness estimation. Eye-tracking data confirmed that the more respondents looked at the Nutri-Score, the better they performed. For NFP, the results were less encouraging, since it either had no effect (compared to a package without Nutri-Score or NFP) or a negative effect (compared to a package with Nutri-Score alone) on performance in the healthfulness estimation exercise. Eye-tracking data confirmed this null or negative effect: In the absence of Nutri-Score, visual attention for NFP did not affect respondents’ answers. However, in the presence of Nutri-Score, visual attention for NFP worsened respondents’ performance, suggesting a cognitive overload issue. Interestingly, we discovered that the positive impact of the Nutri-Score on performance in the healthfulness estimation exercise was not equally large or small for every product: For certain, more equivocal products, the Nutri-Score label had a larger impact than for less equivocal products. For the NFP, we found a negative or null impact on healthfulness estimation accuracy for most products. The only exception to this was a product with an NFP that contained simple and clear nutritional information. In addition to the answers to the research questions, previously unknown effects of age and Nutri-Score familiarity on healthfulness estimation accuracy were discovered. In the next sections, we will discuss all theoretical and practical contributions in detail.

We made several contributions to the literature on FOP and NFP labels. First, we were able to detangle the effects of Nutri-Score and NFP labels on healthfulness estimation accuracy. While most research involving Nutri-Score labels is a comparison between different FOP labels (e.g., traffic light or star rating systems), the current study explicitly envisaged taking the interaction between Nutri-Score and NFP labels under scrutiny, which had not been done until now. This is an important gap in the literature, since consumers from countries where the Nutri-Score has been introduced are exposed to both Nutri-Score and NFP labels in the supermarket. Hence, they will either take one of the labels or both into consideration when making healthfulness estimations. Newman et al. [49], who also study the relation between NFP and FOP labels, suggest that “compared to the more complex (and less accessible) NFP, (…) including reductive (i.e., quantitative) FOP information can generally aid consumers in evaluating the healthfulness of product alternatives”. Newman and colleagues [49] do test whether adding FOP-labels to NFPs could be beneficial; however, they do not test the possible outcome of leaving out the NFP. This might be because the NFP is legally mandatory, but from a scientific point of view, we thought it worthwhile to tease apart the effect of Nutri-Score and NFP labels.

Our findings suggest a suboptimal effect of NFPs given they either had no effect at all (i.e., for products with NFPs, the health estimation performance was not better than for products without), or they had a detrimental effect when combined with Nutri-Score labels (i.e., products with Nutri-Score labels alone outperformed products with Nutri-Score and NFP labels). This result could hint at cognitive overload issues when consumers are exposed to too much or too complex health-related information. This is in line with results from research in which FOP labels are compared with NFP regarding their comprehensibility and perceived cognitive load [10,28]: The more complex NFP is perceived as more cognitively demanding and is least able to increase participants’ healthfulness estimation, while FOP labels are generally perceived as more easy to process.

Our second contribution to the literature introduces the concept of equivocality, or the fact, that some products were more prone to accurate healthfulness estimations than others. Research in the field of FOP labels that considers the (random) effect of products, in particular, is scarce. Our sound analysis of the random factors in our mixed-effects models has allowed us to zoom in on product peculiarities. Interestingly, our findings indicate that for equivocal products, Nutri-Score labels generated the most powerful effect. Put more plainly, products that are more difficult to judge benefit more from the presence of Nutri-Score labels than products that are easy to judge. In our study, the most equivocal products were mocha ice cream (Nutri-Score grade A), rice pudding (Nutri-Score grade B), prawns in garlic butter, and processed lemonade (both Nutri-Score grade E). This implies that the Nutri-Score label affected the healthfulness perception of both products with ‘green’ (A, B) and ‘red’ (E) Nutri-Score grades, which adds up to the conflicting evidence on the impact of traffic light colored labels on healthfulness perception of products. In particular, a recent study found that the Nutri-Score only affected perceived healthfulness in products with ‘green’ Nutri-Score grades (A, B) [22], whereas other studies on the impact of MTL labels found that red lights affect healthfulness perceptions significantly more than green lights [28,36]. We believe that our study provides a possible process underpinning the phenomenon that the Nutri-Score label is more successful for certain products than for others by linking it to equivocality: More than the Nutri-Score grade or the healthfulness of a product, the equivocality of a product determines to what extent the Nutri-Score label is impactful. In sum, a more diversified approach that is adapted to single products, rather than a specific approach for healthy (‘green’) or unhealthy (‘red’) products, could help maximize the potential of FOP labels.

Regarding the impact of the NFPs, it was clear that these labels were not able to constitute a positive effect (not even for equivocal products), with diet coke as the only exception to that observation. The difference between diet coke and other products could be explained by the product’s readily interpretable NFP, which states that the product contains ‘zero of everything’ (0.2 calories, 0 fats, <0.1 carbohydrates, 0 fibers, 0 proteins, and 0.1 salt). This interpretation links to previous research on NFP that confirmed how people can easily interpret NFPs, but have trouble when they need to make more complex calculations [11,39]. For diet coke, the ‘zero of everything’ interpretation might render the NFP easy to judge.

The third theoretical contribution of this study lies in answering the question of whether more visual attention for Nutri-Score and/or NFP make consumers’ healthfulness estimations more accurate. Our eye-tracking analyses confirm the different effects of the Nutri-Score labels and the NFP on healthfulness estimation. The more visual attention towards the Nutri-Score label, the more accurately the product is categorized regarding healthiness. For NFPs, this positive correlation does not hold true: More visual attention is followed by a less accurate healthfulness judgment by the participant. Research that incorporates eye gaze behavior in studying the effectiveness of FOP labels is very scarce. Koenigstorfer et al. [38] do take into account viewing times, but because of the level of detail allowed by the head-mounted eye-tracking devices used, they were restricted to overall viewing times of packages, i.e., they were not able to discriminate between fixations on separate regions of interest. In the present study, carried out with screen-based eye-trackers, we did discriminate between fixations on Nutri-Score labels, NFPs, the remaining parts of the packaging, and outside of the packaging. Our findings show that more visual attention towards Nutri-Score labels (but not towards NFPs) correlates with more successful healthfulness estimations, corroborates the earlier discussion on information overload. For Nutri-Score, long fixations are only indicative of successful processing of the information, while for NFP, longer fixations appear to be resulting from unsuccessful attempts at processing the available information and hint at confusion or difficulty. Nutri Score labels were designed to facilitate a more fluent understanding of health-related information, and our data provide empirical evidence that they indeed allow for more accurate processing.

The interaction between Nutri-Score and NFP labels we observed at the level of estimating products’ healthfulness was also reflected in our eye-tracking data. The detrimental effect of longer fixations on NFP was most prominently the case in the condition in which both NFP and Nutri-Score labels were present. In our view, this is a further argument for the information overload hypothesis raised earlier. NFP alone appears to lead to some kind of confusion because a longer processing time of NFP does not lead to a better understanding (or at least a better healthfulness estimation). Adding the Nutri-Score label only appeared to add to the confusion. More information does not lead to a better performance in this respect. The only exception to this was the already discussed product of diet coke. Longer fixations on the simple ‘zero of everything’ NFP message led to a better healthfulness estimation, which again highlights that message simplicity seems to be key in achieving maximal results.

Finally, this study has several methodological contributions. Some researchers explicitly call for research based on samples that include more variety regarding participant age (and other factors) [22,50]. By including not only students but explicitly aiming at more senior consumers as well, we have reached out to that call. We have managed to compose a diverse sample. For the internal sample composition, we have maximally controlled the spread of eight sociodemographic properties of the participants and eight control questions over the four experimental conditions. This counterbalances the fact that our sample is not representative of the national population. Our findings illustrate the importance of including older participants, as they performed significantly worse than younger participants in the healthfulness estimation exercise. We believe this is not a case of age-grading (i.e., a situation in which young consumers rely on Nutri-Score labels but no longer do so when they grow older), since the Nutri-Score label was only introduced in 2019 in Belgium, but rather the start of a more general shift in acquaintance with the Nutri-Score label. This brings us to the next contribution, which was the impact of Nutri-Score familiarity. This variable significantly predicted health perception as well: Participants that had a better factual understanding of the Nutri-Score label were more accurate in the healthfulness judgments than participants lacking that knowledge. This result provides further empirical ground for Miller and Cassady’s [50] claim that “the more consumers know about nutrition, the more likely they are to consult—and understand—nutrition information on food labels. Lastly, by working with real 2D packages instead of mock products (which is common in research on FOP labels), this study outperforms previous research regarding ecological validity.

Besides the various theoretical contributions, our findings also hold value for policymakers and the industry. In the first place, we have provided further evidence that a simpler system, such as Nutri-Score, outperforms more elaborate systems, such as NFP, in terms of the potential to increase consumers’ correct healthfulness estimations. This result could be relevant in discussions on making Nutri-Score labels more visible or even mandatory, or it could help nutritional professionals in advising consumers on the low hanging fruits towards more healthy food choices. Second, the finding that the Nutri-Score label is more effective for products that are defined as more equivocal (i.e., products that are more difficult to judge regarding healthfulness) is valuable for both policymakers and retailers. If policymakers are informed about the concept of product equivocality, they could raise awareness on common pitfalls in healthfulness estimation, which could eventually help consumers to make more healthy food choices. In a similar fashion, this finding is valuable to retailers as well, since guiding consumers towards more healthy choices fits in Corporate Social Responsibility (CSR) strategies and policies. Retailers could, for instance, highlight the Nutri-Score in Point Of Sale (POS) material placed nearby more equivocal products. This could increase consumer awareness of the Nutri-Score labels for those products, and subsequently increase their visual attention towards those labels. Given that our results show how more visual attention coincides with more accurate healthfulness estimations, POS material might steer consumers towards more healthy food choices.

Next, the finding that familiarity with the Nutri-Score label predicts healthfulness estimation accuracy is highly valuable for policymakers as well: Investing in campaigns aimed at increasing Nutri-Score familiarity could be a highly effective way to steer consumers towards healthier food choices. Overall, we believe that further informing and familiarizing consumers with Nutri-Score labels can help consumers make more healthy food choices. This, in turn, might be an incentive for food manufacturers. Parallel to the notion of ‘teaching to the test’ in education (i.e., teachers concocting a mix of course material so that students would pass standardized tests), the food industry might be tempted into ‘manufacturing to the test’ (i.e., adapting the nutritional composition of their products to meet a healthier Nutri-Score grade).

Of course, we do not turn a blind eye towards the shortcomings of our work. Along the lines of the self-criticism in Becker et al. [37], there is an obvious issue with presenting participants with stimuli that contain NFP and Nutri-Score labels simultaneously. The goal of this simultaneous presentation was to mimic the situation where consumers scrutinize a product by looking both at the front (Nutri-Score) and back/side (NFP) of a pack. However, during natural behavior, consumers are not faced with both labels simultaneously, given the self-evident reality, the labels are printed on opposite sides of a package. Because Nutri-Score labels are front-of-package, and thus, lead to more exposure than NFPs, this might lead us to underestimate the effect of the Nutri-Score and overestimate the effect of the NFP during actual shopping behavior. However, in a real supermarket scenario, a consumer’s decision to select a specific product might also be based on previous experience of using that product without looking at either FOPLs or NFP. Next, we have limited ourselves to measuring healthfulness estimations. A better performance on such an estimation task does not necessarily lead to better food choices (which should be the ultimate goal of Nutri-Score labels). Consumers might treat Nutri-Score labels as a proxy for tastiness (supposing products with unhealthy Nutri-Score labels will be delicious) or overconsume moderately healthy products because they are not ‘all that bad’ [28]. Recent counterevidence [20,30,51] for such undesired side-effects of Nutri-Score labels seems to downplay that risk, however.

To conclude, we consider some avenues for future research. First, this study has emphasized the huge impact of product equivocality on FOP label potential. Therefore, future research should focus on identifying the drivers of product equivocality. Second, we believe that a replication under less controlled experimental circumstances would be relevant. More specifically, a study using mobile eye-tracking devices that are able to discriminate between different regions-of-interest of actual, physically palpable products (rather than 2D onscreen stimuli) could bring research on this topic closer to consumer behavior ‘in the wild’. However, we do acknowledge that such an approach yields other limitations and issues, such as smaller samples, less eye-tracking accuracy, and reliability, or more social desirability bias. Nonetheless, more naturalistic follow-up and replication research remains an endeavor worthwhile. Next, as raised by Hallez et al. [51] in their overview paper, FOP labels interact with many other cues that are present on product packaging. Moreover, visual cues seem to constitute more of an effect than informational cues. In that respect, a study that explicitly addresses the effect of Nutri-Score labels in relation to, for example, sustainability claims, implicit health claims in pictures, or serving suggestions would be a pertinent next step.

## Figures and Tables

**Table 1:** Experimental design, with an example.

		Nutri-Score on Product Visual	
		Nutri-Score Absent (0)	Nutri-Score Present (1)
NFP under product visual	NFP absent (0)	Control 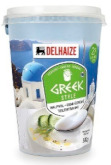	Nutri-Score 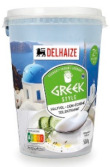
	NFP present (1)	NFP 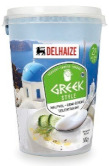 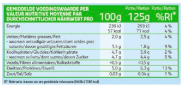	Nutri-Score 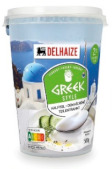 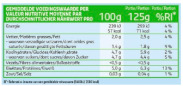

**Table 2:** Sociodemographic characteristics of the general sample (N = 398).

Variable	Total (*n* = 398)	Control (*n* = 102)	Nutri-Score (*n* = 102)	NFP (*n* = 94)	Nutri-Score + NFP (*n* = 100)	*χ*² Test
	*n* (%)	*n* (%)	*n* (%)	*n* (%)	*n* (%)	*p* Value
**Age** (years)						0.540
18–24	222 (55.8)	57 (55.9)	58 (56.9)	51 (54.3)	56 (56.0)	
25–44	90 (22.6)	28 (27.5)	25 (24.5)	20 (21.3)	17 (17.0)	
45–64	63 (15.8)	12 (11.8)	16 (15.7)	15 (16.0)	20 (20.0)	
65 and older	13 (5.8)	5 (4.9)	3 (2.9)	8 (8.5)	7 (7.0)	
**Gender**						0.955
Male	190 (47.7)	48 (47.1)	47 (46.1)	47 (50.0)	48 (48.0)	
Female	208 (52.3)	54 (52.9)	55 (53.9)	47 (50.0)	52 (52.0)	
**Education**	(*n* = 393)	(*n* = 101)	(*n* = 98)	(*n* = 94)	(*n* = 100)	0.341
Low (≤high school degree)	68 (17.1)	20 (19.6)	15 (14.7)	22 (23.4)	11 (11.0)	
Average (=college degree)	94 (23.6)	22 (21.6)	23 (22.5)	20 (21.3)	29 (29.0)	
High (=university degree)	231 (58.0)	59 (57.8)	60 (58.8)	52 (55.3)	60 (60.0)	
**Profession**						0.521
Student or working student	229 (57.5)	59 (57.8)	57 (55.9)	56 (59.6)	57 (57.0)	
Employed	137 (34.4)	35 (34.3)	41 (40.2)	28 (29.8)	33 (33.0)	
Inactive: unemployed/retired	32 (8.0)	8 (7.8)	4 (3.9)	10 (10.6)	8 (8.0)	
**Do you have any expertise in (…) because of your job or education (% yes)?**						
Health	53 (13.3)	17 (16.7)	20 (19.6)	10 (10.6)	6 (6.0)	0.021
Marketing	174 (43.7)	42 (41.2)	46 (45.1)	42 (44.7)	44 (44.0)	0.943
**Shopping frequency**						0.865
Monthly or less	28 (7.0)	9 (8.8)	8 (7.8)	4 (4.3)	7 (7.0)	
A couple of times a month	48 (12.1)	14 (13.7)	15 (14.7)	12 (12.8)	7 (7.0)	
Weekly	135 (33.9)	34 (33.3)	33 (32.4)	29 (30.9)	39 (39.0)	
A couple of times a week	159 (39.9)	37 (36.3)	40 (39.2)	42 (44.7)	40 (40.0)	
Daily	28 (7.0)	8 (7.8)	6 (5.9)	7 (7.4)	7 (7.0)	
**Do you (or someone in your household) follow a (…) diet? (% yes)**						
Medical	61 (15.3)	17 (16.7)	18 (17.6)	11 (11.7)	15 (15.0)	0.677
Weight loss	49 (12.3)	11 (10.8)	21 (20.6)	8 (8.5)	9 (9.0)	0.030
Meatless	104 (26.1)	29 (28.4)	31 (30.4)	22 (23.4)	22 (22.0)	0.478
**Nutri-Score familiarity**						0.540
Is not (very) familiar with the Nutri-Score concept	99 (24.9)	24 (23.5)	21 (20.6)	25 (26.6)	29 (29.0)	
Is (very) familiar with the Nutri-Score concept	299 (75.1)	78 (76.5)	81 (79.4)	69 (73.4)	71 (71.0)	

**Table 3:** Food knowledge, food behavior, and BMI in the general sample (N = 398).

Variable	Total (*n* = 398)	Control (*n* = 102)	Nutri-Score (*n* = 102)	NFP (*n* = 94)	Nutri-Score + NFP (*n* = 100)	One-Way ANOVA
	Mean (SD)	Mean (SD)	Mean (SD)	Mean (SD)	Mean (SD)	*p* Value
Nutrition Knowledge (1–7)	4.79 (1.16)	4.76 (1.20)	4.88 (1.15)	4.77 (1.25)	4.75 (1.07)	0.864
Self-Estimated Diet Quality (1–7)	4.56 (1.31)	4.54 (1.39)	4.60 (1.25)	4.52 (1.34)	4.57 (1.30)	0.977
Nutrition Behavior (1–7)	4.83 (1.17)	4.59 (1.53)	4.73 (1.28)	4.43 (1.53)	4.83 (1.17)	0.198
BMI	23.26 (3.35)	23.20 (3.38)	22.96 (3.57)	23.39 (3.34)	23.49 (3.09)	0.704

**Table 4:** The fixed part of the mixed-effects model explaining AM based on Nutri-Score and NFP, Age, and Nutri-Score familiarity.

	*χ*²	*df*	*p* Value
Nutri-Score presence	47.82	1	<0.001
NFP presence	2.19	1	0.139
Nutri-Score presence × NFP presence	6.68	1	0.010
Age	13.50	1	<0.001
Nutri-Score familiarity	16.39	1	<0.001

**Table 5:** ICC of the random effect terms.

	Stimuli (20 Products)	Participant
Random intercept	0.26	0.08
Random slope for Nutri-Score	0.03	
Random slope for NFP	<0.01	

**Table 6:** The fixed part of the mixed-effects model with a random slope for NFP in the subset of participants exposed to products with Nutri-Score.

	*χ*²	*df*	*p* Value
NFP presence	3.39	1	0.066
Relative fixation duration on Nutri-Score	22.04	1	<0.001
NFP presence × Relative fixation duration on Nutri-Score	0.75	1	0.386

**Table 7:** ICC random effects in mixed-effects model a with random slope for NFP in the subset of participants exposed to products with Nutri-Score.

	Stimulus	Participant
Random intercept	0.20	0.17
Random slope for NFP	<0.01	

**Table 8:** The fixed part of the mixed-effects model with random slopes for Nutri-Score in the subset of participants exposed to products with NFP.

	*χ*²	*df*	*p* Value
Nutri-Score presence	28.56	1	<0.001
Relative fixation duration on NFP	6.53	1	0.011
Nutri-Score presence × Relative fixation duration on NFP	3.75	1	0.053

**Table 9:** ICC random effect terms in mixed-effects model with a random slope for NS in the subset of participants presented with NFP stimuli.

	Stimulus	Participant
Random intercept	0.26	0.07
Random slope for Nutri-Score	0.01	

**Table 10:** The fixed part of the mixed-effects model with a random slope for relative fixation duration on NFP in the subset of participants exposed to products with NFP.

	*χ*²	*df*	*p* Value
Nutri-Score presence	44.40	1	<0.001
Relative fixation duration on NFP	2.75	1	0.097
Nutri-Score presence × Relative fixation duration on NFP	3.97	1	0.046

**Table 11:** ICC random effect terms in mixed-effects model with random slopes for relative fixation duration on NFP in the subset of participants exposed to products with NFP.

	Stimulus	Participant
Random intercept	0.24	0.07
Random slope for relative fixation duration on NFP	0.11	

## Data Availability

The data used in this study are available on request from the corresponding author.

## Appendix A

**Table A1:** Stimuli used in the experiment.

	Nutri-Score A	Nutri-Score B	Nutri-Score C	Nutri-Score D	Nutri-Score E
Beverages					
				
	**Wa**ter (**WA**)	**D**iet **C**oke (**DC**)	**B**lood orange **J**uice (**BJ**)	**F**resh **L**emonade (**FL**)	**P**rocessed **L**emonade (**PL**)
Ice cream					
				
	**M**ocha **I**ce cream (**MI**)	**R**aspberry **S**orbet (**RS**)	**O**range **I**ce cream (**OI**)	**V**anilla **I**ce cream (**VI**)	**I**ce cream **B**ars (**IB**)
Prepared meals					
				
	**V**egetable **C**urry (**VC**)	**M**eat **D**ish (**MD**)	**C**hicken **T**ajine (**CT**)	**T**hai **C**urry (**TC**)	**P**rawns in garlic **B**utter (**PB**)
Dairy products					
				
	**G**reek **Y**oghurt (**GY**)	**R**ice **P**udding (**RP**)	**S**trawberry **Y**oghurt (**SY**)	**O**rganic **C**hocolate mousse (**OC**)	**C**hocolate **M**ousse (**CM**)

## Appendix B

**Table A2:** Scale items nutrition knowledge, self-estimated diet quality, and nutrition behavior.

All Items Were Measured on a Scale from 1 “Does Not Fit with Me at All” to 7 “Totally Fits with Me”	
Nutrition Knowledge	“My knowledge concerning food is limited.” (reversed)
	“I know a lot about healthy food.”
	“I know the basic principles of the food triangle.”
Self-Estimated Diet Quality	“I eat a healthy diet.”
	“Healthy food is part of my way of life.”
	“I try to eat healthy.”
Nutrition Behavior	“Every day, I eat two servings of vegetables (1 serving = a bowl of soup; 3–4 spoons of cooked vegetables; a salad).”
	“Every day, I eat at least two pieces of fruit.”
	“I drink at least 1 L of water every day.”

## Appendix C

The full model explaining the average mistake (AM) in estimating the healthfulness of products was obtained by the following linear mixed-effects model (*r*² = 0.43):

- Random intercept for participants and stimuli,
- By-stimulus random slope for both experimental manipulations (Nutri-Score and NFP),
- Fixed effect terms for the interaction between both experiment manipulations (Nutri-Score × NFP), and for all sociodemographic variables (see Table 2 and Table 3 for an overview).

The fixed part of the model is summarized in Table A3 below (Type II Wald ***χ***²).

**Table A3:** The fixed part of the full mixed-effects model explaining AM.

	*χ*²	*df*	*p* Value
Nutri-Score	46.59	1	<0.001
NFP	2.17	1	0.141
Nutri-Score × NFP	8.35	1	0.004
Gender	0.44	1	0.507
Age	5.38	1	0.020
BMI	0.002	1	0.968
Education	3.08	3	0.379
Professional expertise in Health	1.18	1	0.277
Professional expertise in Marketing	0.47	1	0.492
Shopping frequency	1.77	5	0.881
Nutri-Score familiarity	14.10	1	<0.001
Nutrition Knowledge	1.94	1	0.163
Self-Estimated Diet Quality	2.19	1	0.139
Nutrition Behavior	0.02	1	0.899
Following a medical diet	<0.001	1	0.999
Following a weight loss diet	3.87	1	0.049
Following a meatless diet	0.12	1	0.727

Although the variable Following a weight loss diet is significant in the full model (*p* = 0.049), it is not included as a fixed effect term in the parsimonious model reported in Table 4. We decided to omit this variable since its inclusion caused a computational problem (the model failing to converge with max|grad| = 0.009, which exceeds the preset tolerance of 0.002), resulting in an unstable model.
